# Supplementary material for: Whole-Genome Transcript Expression Profiling Reveals Novel Insights into Transposon Genes and Non-Coding RNAs during Atlantic Salmon Seawater Adaptation
Source: Biology (Basel). 2021 Dec 21;11(1):1. doi: 10.3390/biology11010001 (PMC8772943; doi:10.3390/biology11010001)
Supplement: Supplementary file 1 [file biology-11-00001-s001.zip › Supplementary Figures_revision2_CGE.pdf]

Supplementary Figures

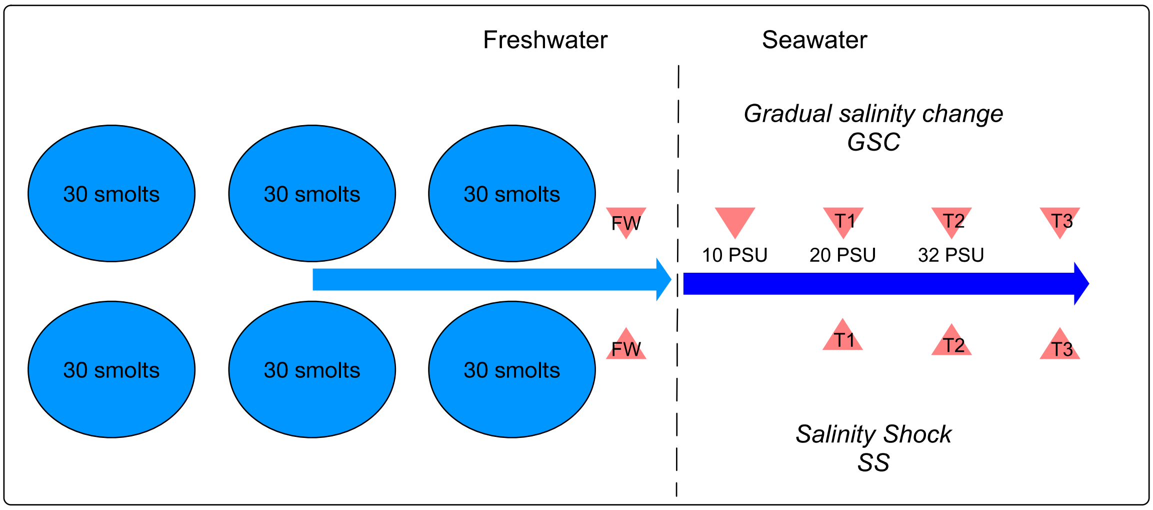

Figure S1

Figure S1. Experimental design diagram showing the GSC and SS conditions for Atlantic salmon smolts (n=30 fish/tank). Sample points (S) are indicated with red arrow. T1-T4 indicate the sample points. For GSC Atlantic salmon group T1, T2, and T3 correspond a week after salmons were at 10, 20, and 32 PSU, respectively. For the SS group, the T1 and T2 are 2 and 3 weeks before salinity shock, and T3 is a week after salinity shock (32 PSU).

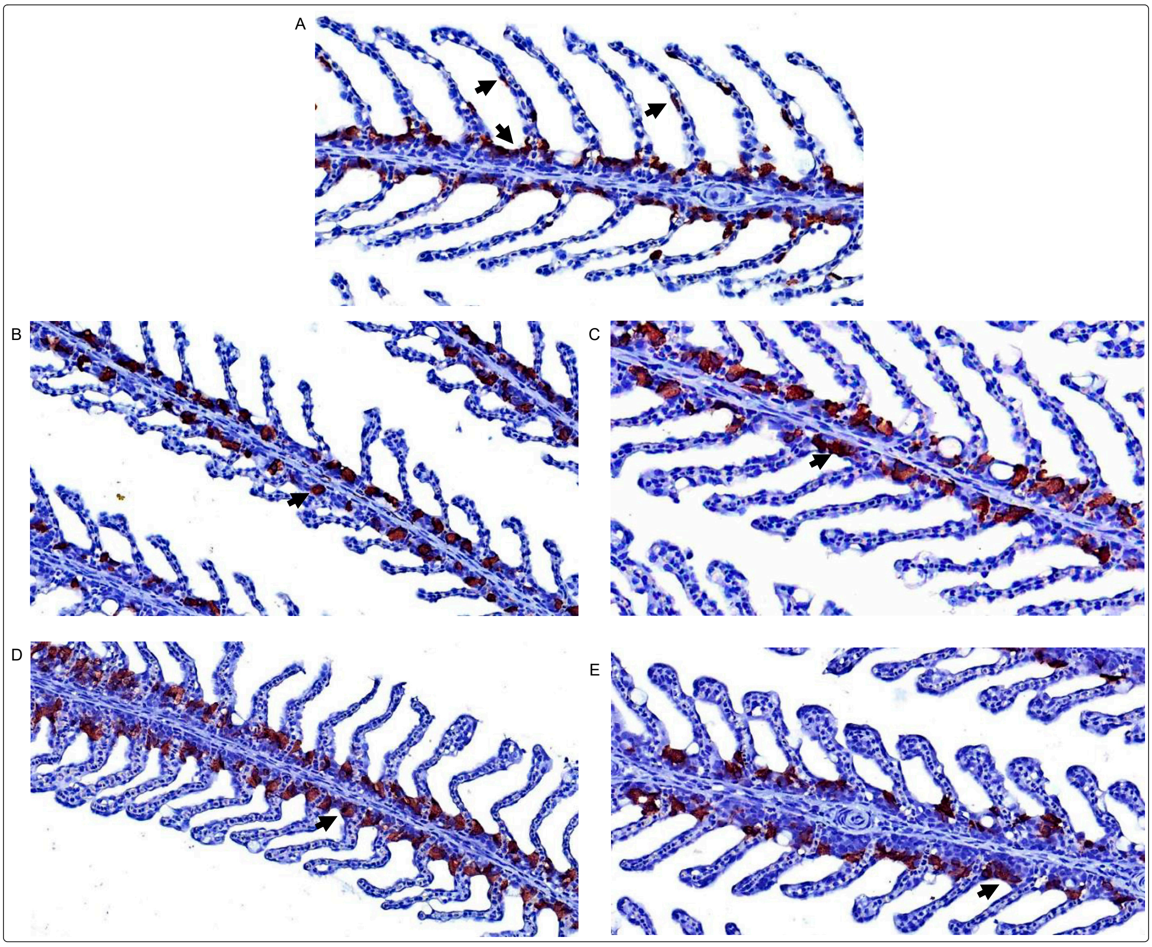

Figure S2

Figure S2. Histochemistry analyses showing the localization of chloride cells in the gill filament epithelium. A) FW gills sample; B) GSC 10 PSU; C) GSC 20 PSU; D) GSC 32 PSU; E) GSC 32 PSU. A) Na+/K+-ATPase positive cells located in the middle region of the superficial interlamellar space and near the lamellar vascular axis. B-C Na+/K+-ATPase positive cell deeper in the epithelium. Na+/K+-ATPase positive cells are indicated with black arrows. Magnification 60x.

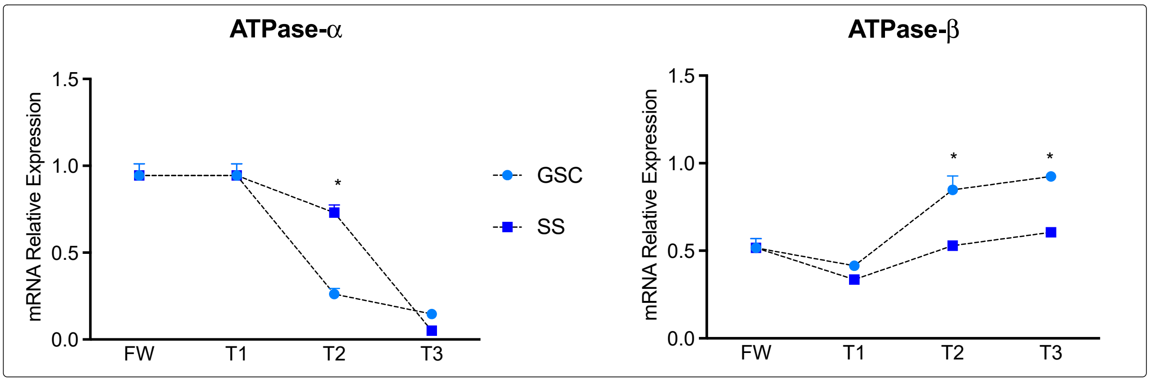

Figure S3. RT-qPCR analysis of ATPase- α and ATPase-β subunits in Atlantic salmon gills exposed to GSC and SS. Significant differences between experimental conditions are indicate with asterisk (p< 0.05).

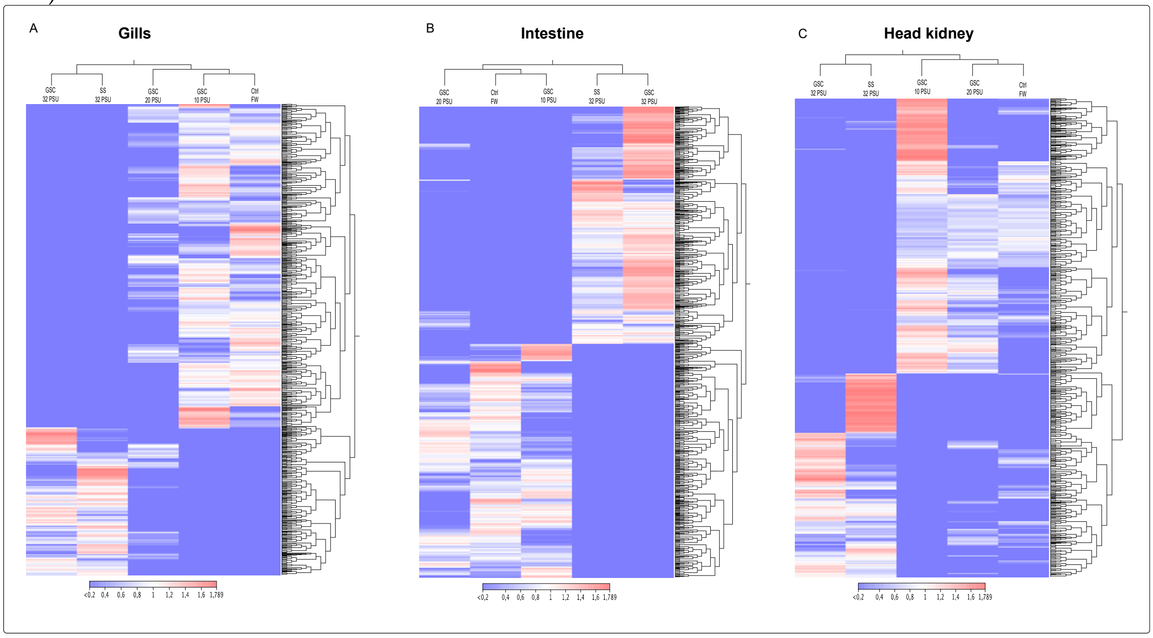

Figure S4. Heatmap representation of Atlantic salmon transcriptome for gills, intestine and head kidney tissues exposed GSC and SS conditions. Red and blue colors represent the gene expression levels from high to low transcription values.

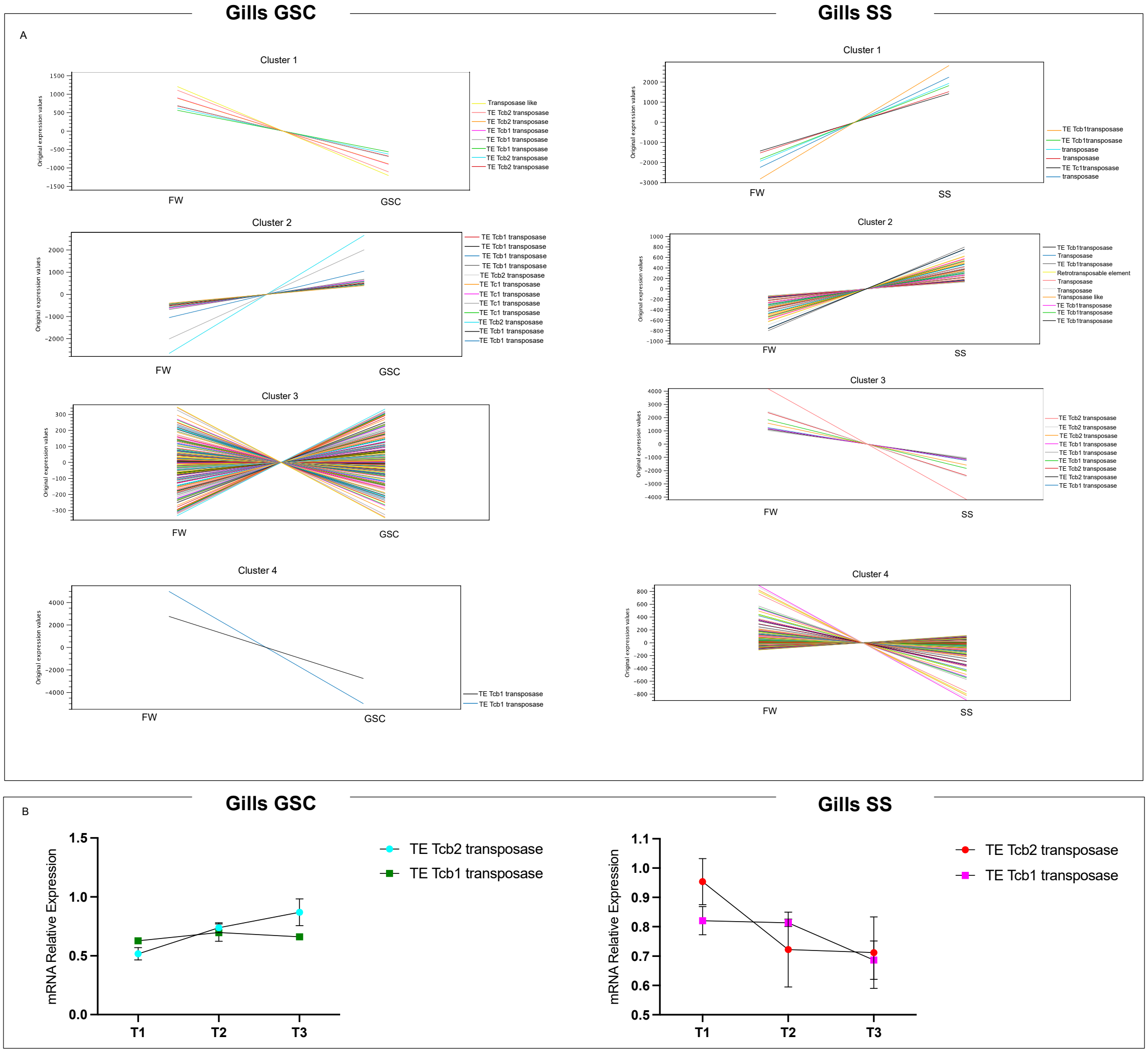

Figure S5

Figure S5. Cluster gene expression analysis of transposable elements (TEs) during SW transfer in Atlantic salmon gills. A) Expression values of TE transcripts in Atlantic salmon gradually transferred from FW to SW (GSC) and from FW to salinity shock (SS), respectively. The four gene clusters were detected by K-means algorithm using TPM values. B) RT-qPCR validation for transposable elements Tcb1 and Tcb2 for GSC and SS, respectively. For GSC Atlantic salmon group T1, T2, and T3 correspond a week after salmons were at 10, 20, and 32 PSU, respectively. For the SS group, the T1 and T2 are 2 and 3 weeks before salinity shock, and T3 is a week after salinity shock (32 PSU). Elongation factor was used as endogenous control for normalize data.

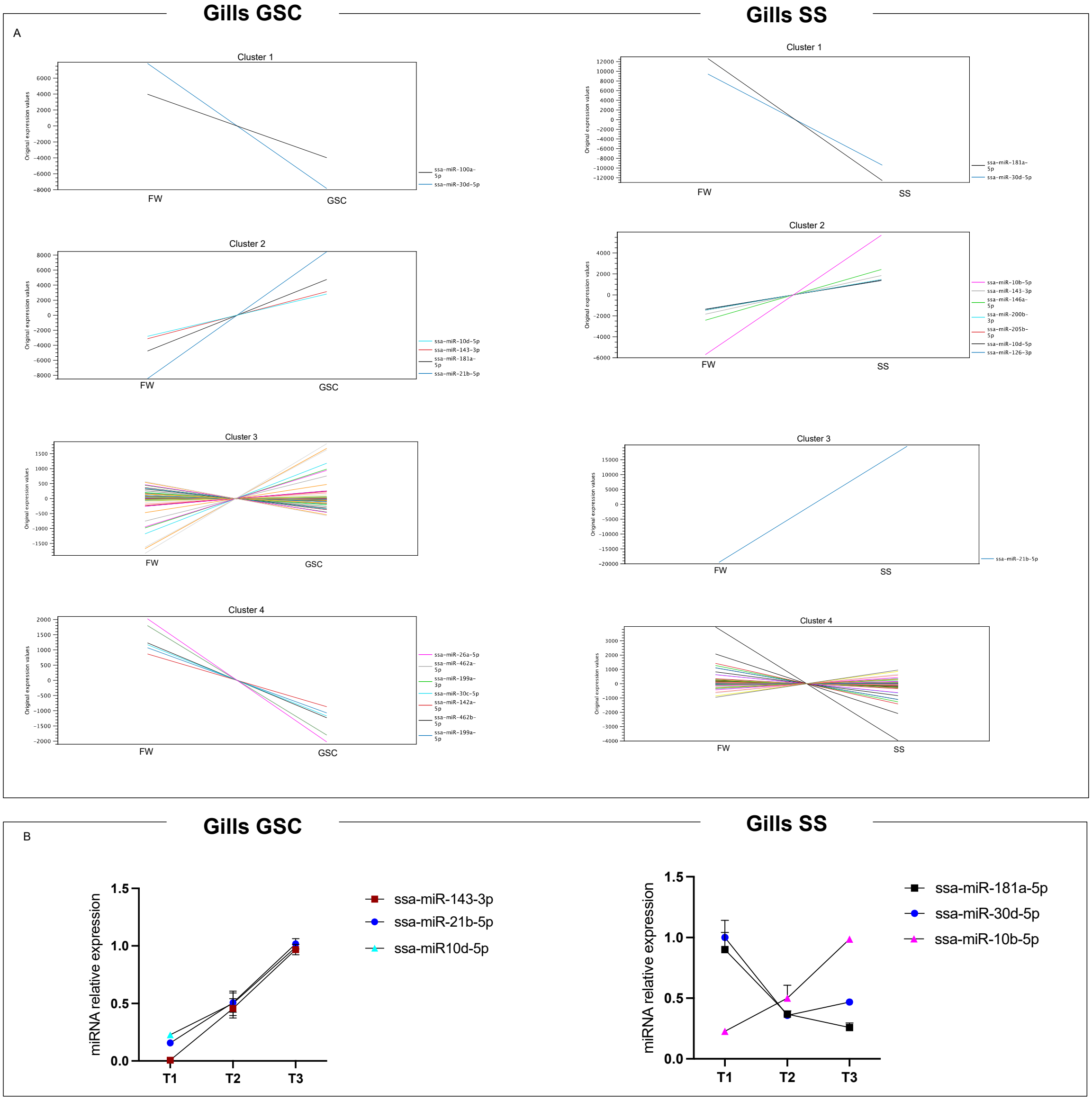

Figure S6. Cluster gene expression analysis of miRNAs during SW transfer in Atlantic salmon gills. A) Expression values of TE transcripts in Atlantic salmon gradually transferred from FW to SW (GSC) and from FW to salinity shock (SS), respectively. The four gene clusters were detected by K-means algorithm using TPM values. B) RT-qPCR validation for ssa-miR-143, ssa-miR-21b, ssa-miR-10d expressed in response to GSC, and ssa-miR-181, ssa-miR-10b and ssa-miR-30d expressed in gills samples exposed to SS. For GSC Atlantic salmon group T1, T2, and T3 correspond a week after salmons were at 10, 20, and 32 PSU, respectively. For the SS group, the T1 and T2 are 2 and 3 weeks before salinity shock, and T3 is a week after salinity shock (32 PSU). Ssa-mir-455-5p was used as endogenous control for normalize data

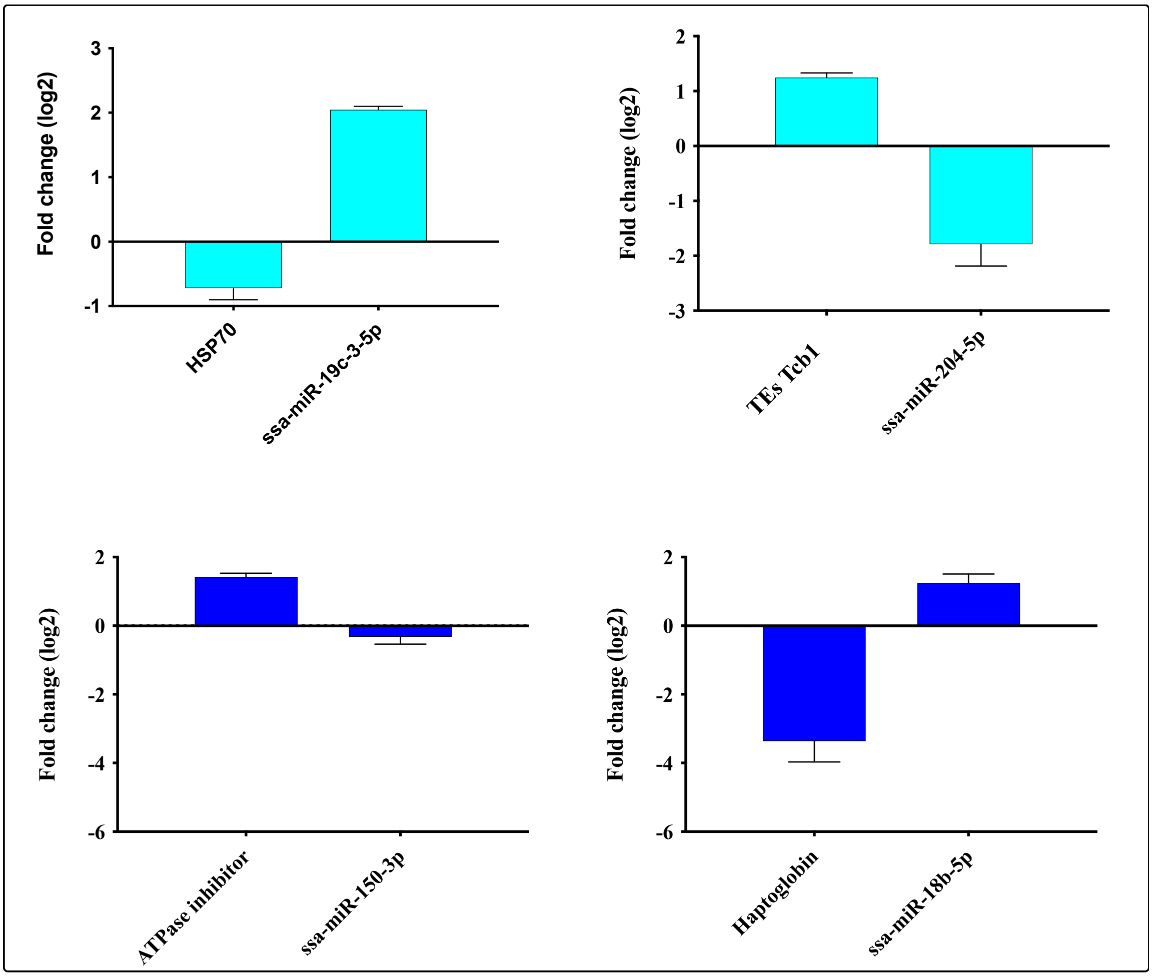

Figure S7. RT-qPCR validation of candidate miRNAs and their putative target gene. Fold-changes (Log_2) of gene expression were calculated using the FW condition as control group. Elongation factor and Ssa-mir-455-5p were used as endogenous control for mRNAs and miRNAs, respectively.
